# Assessing the Vulnerability of Splenectomized Patients to Severe COVID-19 Outcomes: A Systematic Review and Meta-Analysis

**DOI:** 10.3390/vaccines13020203

**Published:** 2025-02-18

**Authors:** Francesco Paolo Bianchi, Massimo Giotta, Andrea Martinelli, Maria Grazia Giurgola, Giulia Del Matto, Elita Mastrovito, Maria Tina Fedele, Giuseppe Manca, Salvatore Minniti, Maurizio De Nuccio, Vincenzo Gigantelli, Silvio Tafuri, Stefano Termite

**Affiliations:** 1 Health Prevention Department, Local Health Authority of Brindisi, Via Napoli 8, 72100 Brindisi, Italy; 2 Surgery Department, Local Health Authority of Brindisi, 72100 Brindisi, Italy; 3 Infectious Diseases Unit, Local Health Authority of Brindisi, 72100 Brindisi, Italy; 4 General Management, Local Health Authority of Brindisi, 72100 Brindisi, Italy; 5 Health Management, Local Health Authority of Brindisi, 72100 Brindisi, Italy; 6 Department of Interdisciplinary Medicine, University of Bari, 70121 Bari, Italy

**Keywords:** asplenia, splenectomy, COVID-19, hospitalization, mortality, lethality, risk factors, vaccination, SARS-CoV-2

## Abstract

Background: Splenectomized/asplenic individuals are at a heightened risk for severe infections due to compromised immune function. However, the impact of splenectomy/asplenia on COVID-19 outcomes remains underexplored. This study aims to systematically review and meta-analyze the association between splenectomy/asplenia and severe COVID-19 outcomes. Methods: Following the PRISMA guidelines, databases including Scopus, MEDLINE/PubMed, and Web of Knowledge were searched for relevant articles published between January 2020 and June 2024. Odds ratios (ORs) and 95% confidence intervals (95% CIs) were calculated for severe COVID-19 outcomes, with a random-effects model being used to account for heterogeneity. Out of 749 identified studies, 4 met the inclusion criteria. Results: The meta-analysis revealed a significant association between splenectomy/asplenia and overall severe COVID-19 outcomes (OR = 1.92; 95% CI = 1.06–3.47). Specifically, splenectomy/asplenia was significantly associated with increased COVID-19-related hospitalization (OR = 2.06; 95% CI = 1.21–3.49), while the association with COVID-19-related death was not statistically significant (OR = 1.52; 95% CI = 0.78–2.99). COVID-19 vaccination is strongly recommended for these patients. Conclusions: Splenectomy/asplenia significantly increases the risk of severe COVID-19 outcomes, particularly hospitalization. The findings underscore the need for vigilant clinical management and targeted interventions for this vulnerable population. Further research is warranted to fully understand the risks and to develop effective guidelines for the protection of splenectomized individuals against COVID-19.

## 1. Introduction

The global spread of COVID-19, caused by the novel SARS-CoV-2 virus, has created unprecedented challenges for public health systems worldwide, particularly in addressing the needs of immunocompromised populations [1]. Among these vulnerable groups, splenectomized or asplenic patients represent a distinct subset at a heightened risk for severe infections, primarily because of their altered immune functionality. The spleen serves as a crucial organ in the reticuloendothelial system, playing indispensable roles in both innate and adaptive immunity. Specifically, it is responsible for the phagocytic removal of opsonized microorganisms, the production of immunoglobulin M (IgM) antibodies, and the clearance of senescent or damaged erythrocytes and other cellular debris from circulation. These functions are vital for maintaining immunological and hematological homeostasis [2].

The absence or loss of splenic function has far-reaching consequences for the immune system, increasing susceptibility to infections and complicating the body’s ability to mount effective immune responses. Patients who have undergone splenectomy, whether due to trauma, hematological conditions, or other medical indications, exhibit a substantially elevated risk of developing overwhelming post-splenectomy infection (OPSI). This rare but life-threatening condition is predominantly caused by encapsulated bacteria such as Streptococcus pneumoniae (responsible for more than 50% of cases), Haemophilus influenzae type b (Hib), and Neisseria meningitidis. The risk of OPSI is particularly pronounced within the first two years following splenectomy but persists at lower levels throughout the patient’s lifetime [2,3,4]. These patients face infection risks that are reported to be tenfold to fiftyfold higher than those seen in the general population. Given their increased risk of infection, asplenic or hyposplenic individuals should adhere to a routine immunization schedule, as recommended by international guidelines [5,6].

While the heightened vulnerability of splenectomized individuals to bacterial pathogens is well documented, the susceptibility to viral infections, including COVID-19, is less clearly established. The mechanisms underlying this vulnerability may include immune dysfunction, alterations in the inflammatory response, and the presence of comorbidities, all of which could exacerbate outcomes associated with SARS-CoV-2 infection [7]. Unlike bacterial pathogens, which can be effectively targeted through preventive measures like vaccinations and antibiotic prophylaxis, the dynamics of viral infections in asplenic populations require further research to clarify their impact on disease severity and progression. Preliminary evidence suggests that splenectomized or asplenic patients may have an increased vulnerability to severe COVID-19 outcomes, such as hospitalization, admission to intensive care units (ICUs), the need for mechanical ventilation, and death. These complications represent the most severe manifestations of COVID-19, as defined by the Centers for Disease Control and Prevention (CDC) [8]. It is hypothesized that immune dysregulation in asplenic individuals may compromise their ability to control viral replication and mitigate the systemic inflammation commonly seen in severe COVID-19 cases. The interplay of cytokine storms, altered T-cell responses, and coexisting medical conditions further underscores the complexity of managing these patients during the pandemic [9].

To address this risk, specific immunization schedules for COVID-19 vaccines have been recommended for splenectomized patients, consistent with international guidelines on vaccination for immunocompromised populations [10]. Clinical studies investigating the safety and efficacy of COVID-19 vaccines in splenectomized individuals suggest that while vaccine effectiveness might be slightly reduced compared to immunocompetent individuals, the safety profile remains comparable. These findings highlight the importance of prioritizing vaccination campaigns and ensuring equitable access for this vulnerable group [11,12,13,14].

Given the paucity of data and the critical need for targeted clinical management strategies, our systematic review seeks to clarify the relationship between splenectomy/asplenia and severe COVID-19 outcomes. Using a rigorous methodological framework, we aim to investigate whether the absence of splenic function significantly elevates the risk of severe disease. The review will also examine potential interventions or preventive measures, including vaccination strategies and post-infection management protocols, that may mitigate these risks. Ultimately, the findings of this review have important implications for the clinical care of asplenic individuals during pandemics and other global health emergencies. Understanding the risk landscape for these patients can inform evidence-based guidelines and ensure that healthcare systems are better equipped to address their unique needs, thereby reducing morbidity and mortality in this high-risk population.

## 2. Materials and Methods

The systematic review protocol was designed and implemented in strict accordance with the Preferred Reporting Items for Systematic Reviews and Meta-Analyses (PRISMA) checklist [15]. To ensure transparency and replicability, the protocol was formally registered in the International Prospective Register of Systematic Reviews (PROSPERO) under the acknowledgment number CRD42024579348. The review question was framed following the Population, Intervention, Comparison, and Outcome (PICO) framework, addressing the specific query: “Is splenectomy/asplenia a risk factor for severe COVID-19 outcomes?”

Search strategy and selection criteria. A comprehensive literature search was carried out using the Scopus, MEDLINE/PubMed, and ISI Web of Knowledge databases to identify relevant research articles. This systematic search was focused on studies published between 1 January 2020 and 15 June 2024. Only primary research articles and brief reports published in English were eligible for inclusion. The search terms used were as follows: (splenectom* OR asplenia) AND (COVID* OR coronavirus OR SARS*) AND (hospital* OR death OR mortality OR lethality OR incidence OR fatality). Studies that did not include epidemiological or clinical data, such as congress abstracts without full texts, reviews, letters, commentaries, or meta-analyses, were excluded. Additionally, research focused solely on unrelated topics, including vaccine coverage or seroprevalence, was not considered.

To ensure a rigorous selection process, the titles and abstracts of all retrieved articles were independently reviewed by two researchers, applying predefined inclusion and exclusion criteria. Any disagreements between reviewers were systematically documented and resolved through consensus after careful discussion. If critical details were missing or unclear, authors of the studies were contacted directly to obtain supplementary information.

Quality assessment. The quality of selected quantitative studies was critically evaluated using the Newcastle–Ottawa Scale (NOS) [16], which is an established framework for assessing non-randomized studies. This tool encompasses seven categories to measure three key aspects of study quality—selection of participants, comparability of groups, and the assessment of outcome or exposure. NOS scores range from 0 to 10, with studies classified as high quality (scores 7–10), intermediate quality (scores 4–6), or low quality (scores 0–3). The assessment was performed independently by two researchers, ensuring objectivity. Disagreements in scores were resolved by discussion.

Data extraction. Two independent researchers extracted data from eligible studies, including their key characteristics, results, and identified variables of interest, into a shared chart. Any discrepancies in extracted data were reconciled by the reviewers through mutual discussion. The extracted information was systematically synthesized and presented to highlight overarching patterns and evidence drawn from the included studies. The systematic review provided a qualitative synthesis, emphasizing the consistency of findings across multiple studies. Special attention was paid to possible strategies for mitigating severe COVID-19 outcomes in individuals with splenectomy or asplenia, with findings summarized in the systematic review results.

Main outcome and pooled analysis. To quantify the relationship between splenectomy/asplenia and severe COVID-19 outcomes, odds ratios (ORs) and their corresponding 95% confidence intervals (95% CIs) were selected as key summary statistics. Hazard ratios (HRs) were converted into ORs to maintain consistency in the meta-analysis framework. This conversion relied on the assumption that COVID-19-related severe outcomes are rare events, thus justifying the approximation [17]. Variance stabilization and normalization were performed using logarithmic transformations of the ORs.

The meta-analysis was conducted using the inverse variance and DerSimonian–Laird weights in a random-effects model, which accounted for between-study variability. Three distinct analyses were performed, as follows:

1. The association between splenectomy/asplenia and overall severe COVID-19 outcomes.
2. The risk of COVID-19-related hospitalization.
3. The risk of COVID-19-related death.

Heterogeneity among the included studies was estimated using a random-effects model and assessed with a *p*-value threshold of <0.05 for statistical significance. The I^2^ index was calculated to quantify heterogeneity and was interpreted using the following thresholds:

- 0–40%: not significant heterogeneity.
- 30–60%: moderate heterogeneity.
- 50–90%: substantial heterogeneity.
- 75–100%: considerable heterogeneity.

To enhance the reliability of the conclusions, the following three sensitivity analyses were performed:

- Sub-analysis considering only high-quality studies (NOS scores 7–10).
- Sub-analysis categorizing populations into general and frail subgroups.
- Leave-one-out analysis to identify and assess potential biases or distortions due to individual studies.

All statistical analyses were performed using STATA MP18 software, ensuring the robust computation of ORs and heterogeneity estimates. Key results were visualized using forest plots to depict pooled estimates.

The results and interpretation of the analyses highlighted key risk factors for severe COVID-19 outcomes associated with splenectomy or asplenia, emphasizing the importance of this information in clinical risk stratification and patient management during the pandemic.

## 3. Results

**Figure 1:**
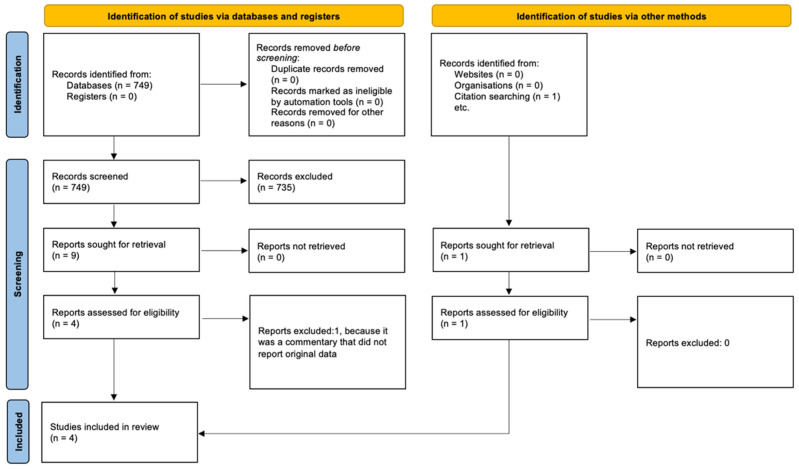
Flowchart of bibliographic research.

Identification of relevant studies. The article selection process was conducted following the PRISMA guidance [15] (Figure 1).

In total, 749 articles were identified from three databases: 23 from ISI Web of Knowledge, 669 from Scopus, and 57 from MEDLINE/PubMed. After removing duplicate articles across the databases and applying inclusion and exclusion criteria, five studies were considered eligible. Among the identified articles, one study was excluded because it was a commentary that did not report original data. As a result, four studies were considered eligible and met the inclusion criteria [18,19,20,21] (Table 1).

Quality assessment. The NOS was appropriately applied to assess the quality of the included quantitative studies, and it was found that 75.0% of the studies were of high quality (Table 1).

**Figure 2:**
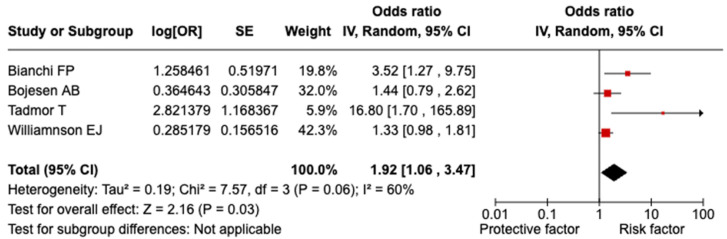
Forest plot of the association between splenectomy/asplenia and overall severe COVID-19 outcomes.

Pooled analysis. A significant relationship was evidenced between splenectomy/asplenia and overall severe COVID-19 outcomes (OR = 1.92; 95% CI = 1.06–3.47; I^2^ = 60.0%; *p* = 0.060; Figure 2). Both the sensitivity analyses considering only the general population and the studies of high quality confirmed the statistically significant relation with a lower heterogeneity (OR = 1.55; 95% CI = 1.03–2.35; I^2^ = 38.0%; *p* = 0.020). Conducting sensitivity analysis by excluding one study at a time did not reveal any significant distortion from a specific paper.

**Figure 3:**
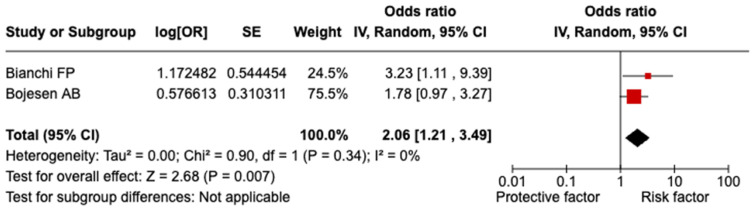
Forest plot of the association between splenectomy/asplenia and COVID-19-related hospitalization.

A significant relationship was evidenced between splenectomy/asplenia and COVID-19-related hospitalization (OR = 2.06; 95% CI = 1.21–3.49; I^2^ = 0.0%; *p* = 0.340; Figure 3). It was not possible to perform any sensitivity analyses due to the small number of included studies.

**Figure 4:**
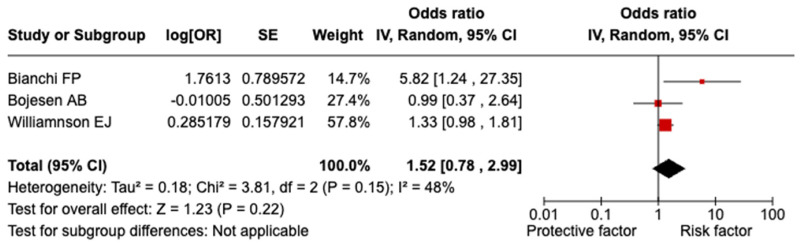
Forest plot of the association between splenectomy/asplenia and COVID-19-related death.

A non-statistically significant relationship was evidenced between splenectomy/asplenia and COVID-19-related death (OR = 1.52; 95% CI = 0.78–2.99; I^2^ = 48.0%; *p* = 0.150; Figure 4). It was not possible to perform sensitivity analysis for population and quality due to the similar characteristics of the included studies. Excluding one study at a time did not reveal any significant distortion from a specific paper.

Strategies to protect splenectomized patients. Several strategies have been suggested by the authors.

Preventive Measures: Splenectomized patients are advised to exercise extra caution and adhere strictly to national public health guidelines to minimize the risk of contracting COVID-19. This includes wearing masks, maintaining social distance, practicing good hand hygiene, and self-isolation [20,21]. Other preventive measures should include prophylactic antibiotics and prompt medical attention for febrile illnesses to prevent severe infections post-splenectomy [18].

Management of Comorbidities: Comorbidities should be carefully managed to reduce the overall risk of negative outcomes in the event of a COVID-19 infection [19,21]. Regular medical check-ups and adherence to treatment protocols for comorbid conditions can mitigate the risk [21].

Monitoring and Support: Healthcare providers should closely monitor splenectomized patients who contract COVID-19 for early signs of severe disease to provide timely medical interventions [19,20]. It is essential to closely monitor these patients for signs of infection and promptly initiate treatment with antivirals that have shown effectiveness in reducing adverse outcomes [19]. Moreover, there is a need to raise awareness among healthcare professionals about the higher infectious risk for splenectomized patients. Ensuring that these patients are properly educated about their condition and the importance of vaccination can help mitigate the risks associated with their immunocompromised status [18]. In particular, Bianchi FP et al. [18] highlighted a key difference between individuals who underwent splenectomy due to trauma and those who had the procedure as part of managing a chronic condition. Trauma-splenectomized individuals are often unaware of their asplenic status and the associated health risks, which may lead them to underestimate their susceptibility to severe COVID-19 complications. In contrast, individuals with an underlying medical condition, such as malignancy, tend to have a higher awareness of their health risks, both personally and through their healthcare providers. This distinction underscores the importance of targeted awareness and education, emphasizing the need for healthcare professionals to recognize and address the different levels of risk perception in these two groups.

Vaccination: All the studies emphasize the importance of prioritizing COVID-19 vaccinations for patients without normal splenic function. Indeed, ensuring high vaccination coverage, including additional booster doses, is crucial for safeguarding this vulnerable group [18,19,20,21]. Despite slightly lower vaccine effectiveness in immunocompromised populations compared to the general population, the vaccines still offer substantial protection [18,19]. Moreover, as reported by Bianchi FP et al. [18], effective communication at the time of vaccination counseling after splenectomy seems to be crucial to ensure long-term compliance with vaccination programs.

## 4. Discussion

Our meta-analysis clearly demonstrates that splenectomy or asplenia substantially increases the likelihood of severe COVID-19 outcomes, with an OR of 1.92 (95% CI = 1.06–3.47). The elevated risk extends to COVID-19-related hospitalizations (OR = 2.06; 95% CI = 1.21–3.49). These results remained robust across sensitivity analyses, including the exclusion of low-quality studies and restricting the analysis to the general population, thereby confirming the reliability of the observed associations. Interestingly, while the link between splenectomy/asplenia and COVID-19-related mortality did not achieve statistical significance, the odds ratio still indicates a potential increase in risk, warranting further investigation. Our findings align with studies on other chronic conditions. A 2022 meta-analysis involving over 900,000 participants reported that COVID-19 patients with chronic liver disease had significantly higher odds of developing severe COVID-19 (OR = 2.44; 95% CI = 1.89–3.16) and experiencing mortality (OR = 2.35; 95% CI: 1.85–3.00) compared to those without liver disease [22]. Similarly, Reyes FM et al. [23], in a meta-analysis of 21,309 patients, found that hospitalized COVID-19 patients with chronic obstructive pulmonary disease faced a significantly higher risk of death (OR = 2.29; 95% CI = 1.79–2.93), whereas no significant difference in in-hospital mortality was observed between COVID-19 patients with and without asthma (OR = 0.87; 95% CI = 0.68–1.10). The impact of COVID-19 on patients with chronic kidney disease (CKD) has also been well documented. A 2021 study found that CKD patients with COVID-19 had a markedly increased risk of mortality compared to CKD patients without COVID-19 infection (OR = 5.81; 95% CI = 3.78–8.94) [24]. Likewise, Raj K et al. [25] demonstrated that adult COVID-19 patients with congenital heart disease were more likely to experience in-hospital mortality (OR = 1.04; 95% CI = 1.04–1.04). A 2022 meta-analysis further highlighted an increased risk of severe COVID-19 outcomes in patients with myocardial injury, hypertension, and diabetes [26].

These findings emphasize the need for vigilant clinical care and pre-emptive strategies to manage the elevated risk faced by splenectomized individuals during the COVID-19 pandemic. The increased susceptibility to severe COVID-19 outcomes in splenectomized or asplenic patients is corroborated by extensive immunological evidence. Splenectomy impairs immune functionality by diminishing the body’s ability to mount effective responses to infections, primarily due to reduced IgM memory B-cell populations and the compromised opsonization of encapsulated pathogens. The SARS-CoV-2 virus exhibits a demonstrated impact on the spleen’s immune compartments. For instance, studies by Lenti MV et al. highlight the significant depletion of IgM memory B-cells in hospitalized COVID-19 patients, directly correlating with increased mortality rates. SARS-CoV-2’s targeted damage to the spleen’s B-cell compartment, particularly its marginal zone, compromises its immunological competence while sparing its red pulp filtering function—a unique dissociation observed in severe COVID-19 cases [9,27]. Further evidence from autopsy studies reveals widespread white pulp atrophy in most COVID-19 fatalities, with 10 of 11 examined cases demonstrating significant structural damage to splenic tissue. Tahtabasi M et al. noted moderate splenic enlargement during the initial phases of COVID-19 infection, which correlates with the severity of COVID-19 pneumonia, as indicated by lung computed tomography findings. Such splenic responses are consistent with cytokine storm syndromes, underscoring the role of systemic hyperinflammation in severe disease progression among asplenic individuals [28,29].

The evidence provided by this review highlights the importance of adopting tailored strategies to mitigate the risks faced by splenectomized individuals during the COVID-19 pandemic. Proactive measures include stringent adherence to public health guidelines, the rigorous management of comorbidities, and regular medical follow-ups to detect early signs of disease progression. Healthcare providers should prioritize vaccinations, including booster doses, for this population, emphasizing the benefits of even moderately effective vaccines in reducing severe outcomes. Clear and effective communication during vaccination counseling is crucial to ensure long-term compliance with immunization schedules. While this study significantly contributes to understanding the risks faced by splenectomized patients, it underscores an urgent need for more targeted research. Future studies should focus on better elucidating the mechanisms underlying the observed associations, particularly regarding COVID-19-related mortality, and explore interventions that could enhance immune responses in asplenic individuals. Larger, multicenter studies that integrate clinical data with immunological and virological findings will be crucial in refining clinical guidelines and developing more effective public health policies for this at-risk group.

This meta-analysis is one of the first comprehensive evaluations of the relationship between splenectomy/asplenia and severe COVID-19 outcomes. A major strength lies in the inclusion of data from multiple studies, enabling a more nuanced assessment of risk for this vulnerable population. By performing rigorous sensitivity analyses, we minimized the impact of biases and methodological inconsistencies across the included studies, ensuring robust and reliable conclusions. For example, our analysis accounted for variations in study quality, population characteristics, and potential confounding factors, providing a clearer understanding of the risks involved. Nevertheless, several limitations should be acknowledged. A primary challenge is the limited number of studies available, reflecting the relative scarcity of research focusing on this specific population in the context of COVID-19. Additionally, significant heterogeneity was observed across the included studies, likely due to differences in study design, geographic settings, and definitions of severe outcomes. While the use of a random-effects model helped address this variability, heterogeneity remains a factor requiring consideration in the interpretation of our findings. The approximation of HRs to ORs, though justified in the context of rare events, may have introduced minor biases, despite our rigorous statistical adjustments [17].

## 5. Conclusions

In conclusion, splenectomy/asplenia is a clear risk factor for severe COVID-19 outcomes, supported by both epidemiological and immunological evidence. The impaired immune function resulting from splenectomy significantly compromises the ability of affected individuals to respond to systemic infections, making them highly vulnerable to complications of SARS-CoV-2 infection. Comprehensive, evidence-based strategies are essential to safeguard the health of this population, including vigilant clinical care, targeted vaccination efforts, and further research to bridge existing knowledge gaps. Protecting splenectomized individuals will not only improve their health outcomes but also contribute to the broader goal of reducing the overall burden of severe COVID-19.

As we move beyond the peak of the pandemic and into 2025 and beyond, healthcare providers must continue to consider the heightened vulnerability of splenectomized individuals. Despite widespread vaccination and natural infection contributing to population-level immunity, this subgroup remains at significant risk for severe disease. Therefore, adherence to updated seasonal vaccination recommendations remains crucial. Healthcare professionals should ensure that these patients receive timely booster doses of adapted COVID-19 vaccines, in line with evolving guidelines, to mitigate risks effectively. Maintaining awareness of these vulnerabilities and implementing proactive prevention strategies will be essential in providing optimal long-term care for asplenic individuals in a post-pandemic world.

## Figures and Tables

**Table 1:** Characteristics of patients with IBD, per group (individuals with IBD vs. general population).

Author	Year	Quality	Population	Country	Sample Size	Study Period	Regression Model
Bianchi FP	2023	h	General population	Italy	2768	March 2020–November 2022	Evaluate the determinants of COVID-19-related hospitalization and death using two multivariate logistic regression models adjusted for sex, age, comorbidities, and COVID-19 vaccine basal routine
Tadmor T	2023	m	Patients with hairy cell leukemia	Israel	218	January 2022–September 2022	Evaluate the determinants of COVID-19-related hospitalization and death using a multivariate Cox proportional-hazards regression model adjusted for age, cytopenia (YES/NO), and cardiovascular disease (YES/NO)
Bojesen AB	2021	h	General population	Denmark	658,923	February–December 2020	Evaluate the determinants of COVID-19-related hospitalization, death, and hospitalization and/or death using three multivariate logistic regression models adjusted for sex, age, and comorbidities
Williamnson EJ	2020	h	General population		17,278,392	February–May 2020	Evaluate the determinants of COVID-19-related death using a multivariate Cox proportional-hazards regression model adjusted for age, sex, BMI, smoking, index of multiple deprivation quintile, ethnic, and comorbidities

## Data Availability

Not applicable.

## Abbreviations

CDC	Center for Disease Control and Prevention
WHO	Word Health Organization
OPSI	Overwhelming post-splenectomy infection
OR	Odds Ratio
HR	Hazard Ratio
